# Patient-centredness in integrated healthcare delivery systems - needs, expectations and priorities for organised healthcare systems

**Published:** 2013-11-28

**Authors:** Christin Juhnke, Axel C. Mühlbacher

**Affiliations:** Institute Health Economics and Healthcare Management, Hochschule Neubrandenburg, Germany; Institute Health Economics and Healthcare Management, Hochschule Neubrandenburg, Germany

**Keywords:** Patient needs, organised healthcare systems, integrated care, factor analysis

## Abstract

**Introduction:**

Patient-centred healthcare is becoming a more significant success factor in the design of integrated healthcare systems. The objective of this study is to structure a patient-relevant hierarchy of needs and expectations for the design of organised healthcare delivery systems.

**Methods:**

A questionnaire with 84 items was conducted with *N* = 254 healthcare experts and *N* = 670 patients. Factor analyses were performed using SPSS©18. The number of factors retained was controlled by Kaiser's criterion, validation of screeplots and interpretability of the items. Cronbach's α was used to assess the internal consistency of the subscales.

**Results:**

Exploratory factor analysis led to 24 factors in the expert sample and 20 in the patient sample. After analysing the screeplots, confirmatory factor analyses were computed for 7-factor solutions accounting for 42.963% of the total variance and Kaiser–Meyer–Olkin of 0.914 for the patients (experts: 38.427%, Kaiser–Meyer–Olkin = 0.797). Cronbach's α ranged between 0.899 and 0.756. Based on the analysis, coordinated care could be differentiated into seven dimensions: access, data and information, service and infrastructure, professional care, interpersonal care, individualised care, continuity and coordination.

**Conclusion and Discussion:**

The study provides insight into patient and experts expectations towards the organisation of integrated healthcare delivery systems. If providers and payers can take into account patient needs and expectations while implementing innovative healthcare delivery systems, greater acceptance and satisfaction will be achieved. In the best case, this will lead to better adherence resulting in better clinical outcomes.

## Background: designing healthcare delivery congruent with patients’ needs?

The consideration of patients*’* needs, expectations and priorities will have a positive effect on adherence and therefore might improve outcomes of healthcare delivery programmes [1,2]. Active involvement of the patient (empowerment) and consideration of patient expectations and priorities can sustainably increase acceptance. Integrated care, coordinated care and disease management are the synonyms for organised healthcare delivery systems that intend to structure the care for a defined population or the care process for a defined indication. One major hurdle to introduce innovative services to the healthcare system is the acceptance of consumers and clinical decision makers. ‘Healthcare systems are challenged to effectively meet the wants and needs of patients by tailoring interventions based on each person's […] preferences as well as personal and social context’ [3]. As experts act in the interest of the people in need, patients’ and experts’ acceptance of features of integrated care systems is a prerequisite to become a standard feature of today's healthcare systems. Innovative services and organisations aim to achieve higher quality care, lower costs and greater patient satisfaction. The Institute of Medicine report ‘Crossing the Quality Chasm’ [4] emphasises that health decisions should be customised based on patients’ needs and values. Porter and Teisberg state ‘healthcare is on a collision course with patient needs and economic reality’ [5]. In health policy terms, this refers to services ‘closely congruent with, and responsive to patients’ wants, needs and preferences’ [6]. Authors conclude that the problem is not a lack of knowledge, nor is it the people's unwillingness to spend money. Rather, the difficulties lie in the understanding of peoples’ priorities.

### Patient-centred healthcare delivery systems

The most powerful structural innovation in healthcare will be based on a paradigm shift - patient-centred healthcare systems. Despite this hypothesis, policy-makers and healthcare professionals conceptualise most, if not all, healthcare delivery systems, with little involvement of the general public. Moreover, the published literature does not clearly specify the relative importance patients place on certain characteristics of integrated care. The acceptance of a (integrated) care programme is based on patients’ evaluation of the properties and attributes of the services provided. Designing services that are sensitive to patients’ needs in the context of limited resources may require policy and decision-makers to choose between several design characteristics. This is one of the biggest problems that policy and decision-makers are facing in the coming years and raises questions of how healthcare delivery systems should be organised and services provided. Moreover, meeting expectations on a range of attributes may be difficult within the constraints of limited budgets; this has led to interest in methods for assessing priorities. In the case of integrated delivery systems, patient value will be assessed in terms of a set of patient-relevant properties of organised delivery systems.

### Research question

The study was intended to identify potential needs, expectations and priorities of patients towards organised healthcare delivery systems. The aim was to identify characteristics, which determine utility, motivation and quality of care from the patients’ perspective, and the healthcare providers, payers and other experts’ point of view. The paper addresses the following research question: Which dimensions best describe patients and experts’ priorities towards a better organisation of health care delivery systems? Which characteristics and dimensions increase patients’ utility from a consumer and expert point of view? The research results are intended to be used in preventative, curative and rehabilitative sectors of patient care and to help design patient-centred healthcare services.

### Decision-making context: What are needs? What is patient-centredness?

#### Patients’ needs

In general, need is understood as ‘a feeling of deficit with the desire to relieve this’ [7]. Removal of a deficit is known as satisfaction of need. With respect to this definition, priorities can be seen as the order of the removal of the deficits. A term closely linked with the concept of need and priorities is that of utility. Demand, resulting from needs, based on (expected) utility is to be seen as the results of successive sub-processes and can be considered an intellectual sequence of economic behaviour. From an economic viewpoint, needs represent a significant component of motive since they are responsible for the processes of economic action and can thus be used to explain the behaviour [8,9]. If patient priorities are taken into account adequately, it is safe to assume that this will increase acceptance due to an increase of satisfaction with, and adherence to, clinical treatments and public health programmes. For this reason, needs are the starting points for an analysis of the motivation for utilisation behaviour and patients acceptance of innovative healthcare delivery systems. Even though these terms are commonly used they still lack a uniform definition. In order to analyse the needs and expectations of patients for these kinds of care delivery systems the terms need to be defined.

#### Organised healthcare systems

‘Organised healthcare systems’ can be seen as a collective term for many different forms of healthcare: integrated care, coordinated care, multidisciplinary approaches or general practitioner-centred care, that ensure trans-functional, patient-centred, rational provision of healthcare services across the whole continuum of health needs [10,11]. As such, the term ‘organised healthcare networks’ covers all the networks which constantly organise and/or provide healthcare to a defined population group and bear both medical and financial responsibility for the healthcare [12].

#### Integrated care

Integration represents a unified methodology set incorporating the financing, administration, organisation and provision of services, in order to establish coordination and cooperation between the various sectors of the healthcare system [13]. Integrated care ensures trans-functional, patient-centred, rational provision of healthcare services across the whole continuum of health needs [10,11].

#### Coordinated care

The term ‘coordinated care’ has to date no unified, generally valid definition. Instead there are many attempted definitions of the term. Bodenheimer defines coordinated care as the ‘conscious integration of processes of patient care between two or more actors involved in the treatment in order to enable or facilitate appropriate care of the patient’ [14]. The central element of many attempts to define coordinated care is patient-centredness and the focus on specific target groups rather than being population-oriented [15]. Chen et al. distinguish two forms of coordinated care in their definition: case management and disease management [16].

## Method and study design

### Study design

A multilevel methodological approach was chosen: in addition to the systematic literature research complementary, qualitative and quantitative methods were used to combine patient's perspective (bottom-up) as well as evidence generated from literature research (top-down). The goal of this mixed-method-approach was a comprehensive data collection and the documentation of all potentially relevant properties and characteristics of integrated care programmes.

The combination of qualitative and quantitative methods of examination and analysis enables the reproducibility of measuring procedures, for example, for the purpose of adaptation to various cultural contexts [17–19]. The aim was to build the collection and analysis of needs and expectations on the psychometric structures and dimensions of the needs.

### Literature research

A literature search carried out in September 2010 on empirical studies in the field of ‘patient-centred coordinated care’ produced a very differentiated study situation. The systematic research showed that up to then only a few studies were available which document patient preferences in integrated care using discrete-choice experiments [20,21]. On the other hand, there are a large number of empirical studies which examine or describe the significance of various care characteristics to patients.

The literature search covered primarily German and English language sources in renowned databases, particularly PubMed, MEDLINE, SpringerLink and Web of Science. In addition, the publication data of established organisations such as the Cochrane Collaboration, the Commonwealth Fund, the Agency for Healthcare Research and Quality or the World Health Organization were searched for relevant entries.

The keywords used were ‘integrated care’, ‘coordinated care’, ‘patient-centred (coordinated) care’, ‘patients’ perspective’, ‘preferences’, ‘patient priorities’, ‘conjoint analysis’ and ‘discrete-choice’ and their various combinations as well as spellings.

In addition, case studies from the Commonwealth Fund ‘Case Studies of Organised Delivery Systems’ were included in the analysis as successful care models [22].

For the initial evaluation, a total of 662 sources were then examined in a systematic analysis on the basis of title, abstract and full text as to their relevance for this study. The references included were those which analysed either the patient's or doctor's perspectives or their expectations or priorities on (aspects of) coordinated/integrated care, or which presented the success factors of organised healthcare networks in general. References were excluded when meeting at least one of the following criteria: language not English or German, publication date before 1990, purely clinical/medical articles describing therapy procedures, topic is economic evaluation of coordinated healthcare approaches, descriptions of the controlling effects of financial incentives in various countries.

**Figure 1.**
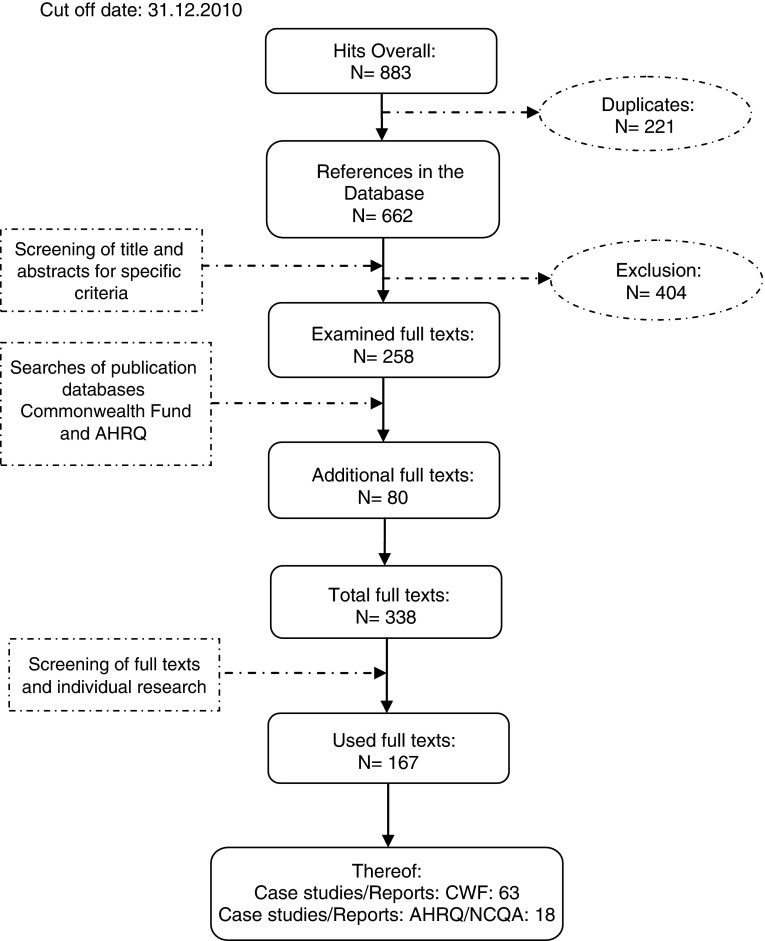
Methodology of the literature search.

A total of 338 references remained. In the course of screening the full texts further references were excluded, whilst other references were added by individual research. Finally, 167 articles were included. The procedural method of the literature search is presented in Figure 1.

### Qualitative research

The study was based on the hypothesis that the motivation for the utilisation of and satisfaction with care programmes can be increased as well as the long-term treatment success of supply if the offers are based on the needs of patients.

The explorative needs analysis was conducted using the design-thinking method. For this, patients were interviewed directly and viewed from a third-party perspective. This method takes physical and psychological as well as social and cultural factors into consideration. Design-thinking enables analysis of the needs patients formulate verbally and non-verbally. Through intense observation and projection into the actual experience of utilisation, the underlying motives and motivations of behaviour become clear. In the design-thinking method, spontaneous interviews are supplemented by targeted in-depth interviews with various persons in the typical user environment (healthcare system facilities). Some of the interviews are conducted as an accompaniment to specific experiences in order to obtain observations as direct as possible, original statements and unfiltered interpretations [23].

The interviews and focus groups took place mainly in the day-to-day and communication environment of those affected. The focus was on people with chronic diseases. In addition, relatives, non-patients, experts and employees from healthcare facilities services were surveyed (*N* = 22). After the interviews with the design-thinking method therefrom derived protocols were analysed and tested for significant features. As consequence a list of potentially relevant characteristics of healthcare delivery systems emerged. This list was then compared to that developed within the literature analysis and supplemented, if necessary.

### Quantitative research

The qualitative results of the design-thinking, the focus groups as well as the literature research should be assembled to extract only those items relevant for patient needs and expectations.

**Figure 2.**
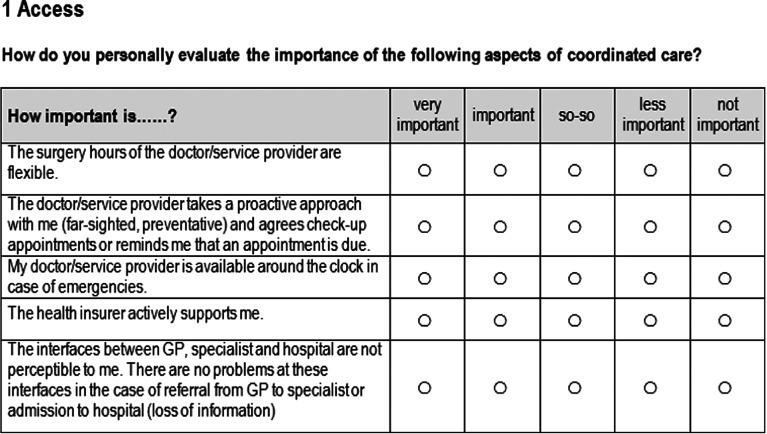
Example of 5-point Likert scale in the quantitative survey.

Care delivery-related items were assembled in a questionnaire to structure them. Healthcare experts (care provider, payer and healthcare decision maker) as well as patients were ought to rate these items according to their individual meaning using a 5-point Likert scale in paper-and pencil-based surveys. The scale ranged from ‘very important’, ‘important’, ‘so-so’, ‘less important’ to ‘not important’ (Figure 2). The patient surveys as well as the expert version consisted of the same care-related items. Next to the rating the questionnaire included a socio-demographic part.

Patients were surveyed in 12 medical practices in three German federal states between June and October 2010. Healthcare experts were surveyed between April and June 2010 at three international health conventions. The patients were made aware of the survey, both directly and through flyers and asked for their participation. The sampling strategy of the participants in the patient survey was based on the inclusion and exclusion criteria age (18–80 years), language skills (German), cognitive capability, health status (no serious, acute illnesses or pain) and interest in participation. The recruitment of experts was carried out by a direct address to the people by the authors as well as by research assistants during the three congresses.

Psychometric testing and dimensional structuring of the care-related items was affected by means of exploratory and confirmatory factor analysis using SPSS®18. A correlation analysis was conducted to verify whether the items are depending on each other or not. Those items highly correlating were identified by the means of factor analyses and outlined in diverse dimensions. Factor analysis is a multivariate method that is primarily used in exploratory studies to investigate the interrelationships between many variables. The primary goal of factor analysis is to structure a larger set of variables, and to analyse which of the observed variables can be combined in common information (factor) and which variables are inseparable [11,24]. The development of factor analysis has been driven in different directions, there is now not a fixed method of analysis, but rather a variety of approaches to factor analysis. As used in this project, principal component analysis is currently the most common method [25].

#### Sample size

The literature provides no clear indication of how large the sample must be for a successful factors analysis. However, the ratio of persons interviewed and tested variables has established itself as an appropriate rating scale. In practice ratios of less than 2:1 up to 10:1 are described [25].

In the present study, a ratio of respondents to variables of 3:1 was also adopted as a target. The results showed a ratio of 2.89:1 in the expert survey and 7.61:1 in the patient survey.Another means of verifying the sample quality is the Kaiser–Meyer–Olkin coefficient. The Kaiser–Meyer–Olkin coefficient is based on the partial correlations between the variables and provides information on whether the variable selection and sample size are suitable for factor analysis. The Kaiser–Meyer–Olkin value is classified as follows: <0.50: incompatible with analysis, 0.50 to 0.59: bad, 0.60 to 0.69: standard, 0.70 to 0.79: medium, 0.80 to 0.89: good and >0.90: very good [26].

## Results

### Qualitative research

The qualitative results of the design-thinking, the focus groups as well as the literature research were assembled to extract only those items relevant for patient needs and expectations.

As a result, 84 care delivery-related items (e.g. ‘I can order remedies, therapeutic appliances and medicinal products from anywhere’ or ‘The doctor/service provider provides interdisciplinary, all-encompassing health care’) were extracted and assembled in a questionnaire for the quantitative study phase to structure them. Healthcare experts as well as patients rated these items according to their individual meaning using the 5-point Likert scale.

### Quantitative research

The psychometric analysis of the rating aimed at the determination of the most relevant (‘main’) needs dimensions. By means of a correlation analysis the extent to which different characteristics are independent was also evaluated. Characteristics that are highly correlated with each other were determined by an exploratory factor analysis using varimax-rotation, structured with reliability analyses and combined into dimensions.

#### Experts

Overall *N* = 254 experts could be acquired, who were predominantly male and German and working in healthcare management (Table 1).

The exploratory factor analysis led to 24 factors with Eigenvalue > 1, Kaiser–Meyer–Olkin of 0.730 and 71.357% of total variance. Missing values were replaced by mean values. After analysing the screeplots and qualitative results, confirmatory factor analysis was computed for a 7-factor solution (load > 0.4). This accounted for 38.427% of the total variance and with Kaiser–Meyer–Olkin of 0.797. Cronbach α reliability coefficients were computed for each of the sub-scales and ranged between 0.836 and 0.715. Based on the existing literature and the analysis conducted, coordinated care could be differentiated into seven dimensions: individual care and participation in social life; professional and interpersonal care; access; data, information and knowledge transfer; continuity and coordination, prevention and health promotion as well as service and infrastructure [27].

The results of the factor analysis can be found in Table 2. Displayed are the identified factors with reliability coefficients as well as the items and the three highest loading items.

#### Patients

Patients’ elicitation was conducted in three German federal states. Overall *N* = 670 patients took part in the survey (mean age: 48.47) and could hence be included in the analysis. The socio-demographic analysis is presented in Table 3.

To identify the dimensional structure within the patient survey all 84 therapy-related items underwent exploratory factor analysis (main components) with varimax-rotation. An unrestricted analysis resulted in a Kaiser–Meyer–Olkin of 0.914 with 20 factors with Eigenvalue > 1 and a 63.462% of total variance.

The screeplots showed plausibility for a limitation on 4–8 factors. Following a content analysis of the results and a discussion in an expert-panel, the 7-factor solution was confirmed to be most reliable. Within this 7-factor analysis, listwise deletion (exclusion of cases if any single value was missing) and suppressed coefficients were used. Following the deletion of incomplete cases, 453 cases remained. Based on the Kaiser–Meyer–Olkin = 0.914 and a variance of 42.963%, the sample was valid for factor analysis.

Within the 7-factor solution, Cronbach α reliability coefficients ranged between 0.899 and 0.756. In order to increase Cronbach α items with a load < 0.4 were excluded from further analysis (Table 4).

## Discussion

In order to create or guarantee patient-centred care, it is essential to ascertain patient expectations, priorities and needs. For this it is primarily the opinions of the patients themselves which are relevant, although the analysis of expert views is also important.

The study has shown that the views of patients and doctors are not entirely different. In the rating survey, the parameters of professional and interpersonal care are by far the most important in the view of both patients and doctors. It is striking that of the ten items evaluated most highly by patients and experts, seven are identical.

In the factor analysis, a similar picture emerged. The doctor and patient perspective is similar, but not identical. For both seven factors emerge, but with differences in their sub-dimensions. The greatest correspondence of experts and patients is in the need dimensions ‘Coordination and Continuity’ and ‘Service and Infrastructure’, which are completely identical.

In order to develop a common model of need dimensions in organised healthcare delivery systems the initially described results were further analysed and discussed. The overall aim of this process was the development of an all-encompassing model that ascertained expectations of both patients and experts.

The generated model from patients and experts survey can be summarised as shown in Table 5.

The seven factors that made up the final model of needs dimensions are described in the following sections.

### 

#### Access

The access to facilities, organisation and services is the precondition for utilisation of healthcare services. Both geographical and timely access to healthcare services should be based upon the needs and expectations of the patients affected [28–31].

Geographical access relates in real terms to the close proximity of general practitioner care, specialist care within a reasonable distance and hospital care in line with patients’ needs.

This covers both the distance to health professionals and technical and clinical facilities, for example, hospitals [32].

‘Timely access’ tends to cover the organisational arrangement of entry into the healthcare system. This covers in particular opening hours, availability of appointments and waiting times both for an appointment and the time spent in the office [28,31,33].

Access to healthcare services can be made more difficult by various ‘access barriers’. An initial barrier can exist in financial aspects. Supplementary payment rules may be one reason for not visiting a doctor. Particularly in healthcare systems in which many services must be paid for by members themselves, it is often not possible for low earners to finance these services or to pay insurance premiums [34].

#### Infrastructure and service

‘Infrastructure and service’ refers mainly to two areas: the physical (structural) conditions of healthcare facilities and the staff and associated characteristics. The structural conditions and resources secure the healthcare, but do not guarantee it. They do affect care indirectly and also have an influence on the quality of the services provided in the facility. If required resources, medical technology or staff resources are not available, this affects the quality or leads to a loss of services [35].

‘User-friendliness and accessibility’ concerns unhindered access to the healthcare facility and furnishings in line with patient needs [36,37]. This refers above all to the infrastructural situation. Adequate parking and public transport within the facility can significantly influence patients’ perception of this needs dimension. The unhindered accessibility of healthcare services plays an essential role in the care of physically handicapped persons in terms of the availability of healthcare services to them.

‘Furnishings and facilities’ describes the equipment, fittings and service aspects within the various facilities of the healthcare system. Achievement of a high standard in the treatment of specific diseases requires adequate preparation or adaptation of a facility [36]. This also includes ensuring that it is easy to find one's way around the facility [31,33,38].

Besides the furnishing and service aspects, from a patient perspective a friendly atmosphere between the staff and between staff and patients is important and affects the nature of service provided [35]. Unlike ‘interpersonal care’, however, in this sub-dimension of ‘personnel’ the doctor–patient relationship is not the focus of attention. This sub-dimension describes the social and psychological interactions outside the direct doctor–patient communication [35].

#### Data and information

Especially in healthcare system it is true that data are not necessarily information, and information is not necessarily the required (deciding) knowledge. Data (in the sense of individual details of a condition in measurable quantities) only do make sense to patients when put in context - that is they are contextualised into ‘information’.

To actively involve a patient the organised care network should make ‘Health Information’ available that is accurate and of high quality [39–41]. In this context, it is particularly important to patients that they receive the information relevant to them presented individually and in a patient-friendly form [31]. The aim is to strengthen the decision-making competence and cognitive abilities of patients by specific knowledge and to designate the quality of the information available (exclude abuse).

Central in terms of ‘Patient Data’ is the transparent and safe collection and storage of data. From the patient perspective, it is important to ensure that he or she has access to his own patient data, has free disposal over it and that data security is ensured. Transparency, availability and security, so that patients have access to and control over their own medical data when required without difficulties [42].

Finally, the availability and quality of ‘Performance Indicators’ are other important factors in this dimension. The offer of information about the healthcare provider, his or her specialisation or quality certificates as well as the transparent presentation of the range and scope of the healthcare services on offer have shown to be relevant to patients [31,41,43].

#### Professional care

The dimension ‘Professional Care’ covers clinical care [35] and describes the medical and specialist care [44] provided by the various healthcare providers in the course of organised healthcare [45,46] but also measures to guarantee (medical) quality assurance and patient safety. A system of healthcare delivery is seen as qualitatively high if designed to be effective, safe, coordinated and patient-centred [47].

One aspect of this dimension is information transfer or ‘Patient Education’. In the transfer of knowledge by the healthcare provider the issue is how the patient is educated so that his or her competences are increased, patient self-determination is retained and, as far as possible, the patient assumes responsibility for his health - with the goal of enabling to the necessary decisions to be made. The sub-dimension ‘Competences of the Healthcare Provider’ is characterised by professional patient care within the care concept and its adaptation to the needs of the patients. This takes account not only of the specialism of the treating doctors but also the competence and experience of the doctors. Central issues here are whether and in what way staff qualifications as perceived by the patients have an effect on treatment success.

In recent years, ‘Medical Quality Assurance’ has assumed increasing significance. Medical care is expected to correspond to the latest standards of medicine (evidence based), be appropriate and not be performed unnecessarily [42]. This means that all healthcare providers bear responsibility for excess, inadequate or incorrect healthcare.

#### Coordination and continuity

Coordinated care is on the one hand the long-term planning and management of the care of a patient and on the other hand an integrated care programme which must be coordinated between the healthcare providers and sectors involved [22,48]. Continuity of care actually means that there is a guaranteed transition from one healthcare provider to another (see Care Transition Intervention [49]), or that the patient has a constant contact person who is responsible for the coordination of services (see Chronic Care Model [50] or Medical Home Concept [51,52]). ‘Interdisciplinary Care’ describes interdisciplinary trans-specialist collaboration between various healthcare providers and/or social organisations within a healthcare sector (sometimes referred to as horizontal integration) [53]. The framework encompasses linked and consistent patient care provided by healthcare providers which remain constant as far as possible and that work together with other specialists within one healthcare sector [54]. This is intended to ensure that patients receive comprehensive care within the familiar framework based on their needs (see Patient Empowerment) [28,39].

Ideally, besides interdisciplinary care there should also be ‘Coordination of Care’ via several healthcare providers representing various levels of care [55]. In practice, this describes the integration of healthcare processes which represent a trans-sectoral provision of services, for example, hospital, out-patient care and rehabilitation [53]. The continuous care of a patient or a population group includes not only management of acute care but also the organisation of prevention and after-care consultations.

In order to optimise coordination and continuity in patient healthcare provision, it is essential that all those involved contribute to a better exchange of information and thus to a consequent improved communication culture. ‘Informational Continuity’ describes the continuous and up-to-date transfer of information on test and examination results and mutual notification. For designing patients’ healthcare effectively within a network of coordinated care the exchange of information or communication thus has a considerable influence on the quality and cost effectiveness of the healthcare delivery [56].

#### Interpersonal care

Interpersonal care is seen as one of the most important characteristics of good healthcare services in the sense of patient-centred care [57]. Interpersonal competences are also an indicator of therapeutic competence. Deciding criteria for successful treatment from the patient perspective are perception of their individual needs, a high level of sensitivity, comprehensive professional information and the observance of psycho-emotional relations [58].

The sub-dimension ‘Patient–Physician Relationship’ focuses on the non-medical care of the patient. This concerns interpersonal care, that is, attitude, approach and behaviour towards the patient. This covers in particular aspects such as attentiveness, honesty and openness during treatment. A personal relationship to a known and constant contact person is decisive for patients [59] and effects compliance and adherence [57,60].

The aim of ‘Patient–Physician Communication’ consists of ascertaining the ideas, values and priorities of the patients and integrating these into the healthcare communication [45]. An important ability in the field of interpersonal care is the healthcare provider's ability to communicate [33,39] which should enable honest, open and understandable communication in which the patient also has the chance to express his opinion [33]. Empathy and attentiveness, so that patient can discuss the health problems, also play an important role [47].

Patients fluctuate between the need for self-determination and the desire for the provision of clear paths and terms of references by the medical professionals responsible for their care (experts). Individualisation and ‘Patient Empowerment’ are an expression of patient-centredness and a decisive criterion for success. The intention is to give patients more responsibility for themselves and their healthcare, and involve them in decision-making for therapies (shared decision-making) [31,33].

#### Individualised healthcare

The institutions of the healthcare system are intended to consciously consider the individual and social context of the patients and involve them more intensely in diagnostics and therapy [33]. Social interaction, the maintenance of social contacts and the support of relatives or self-help groups have a positive effect on the success of treatment [61]. ‘Individual Management’ by the patient describes primarily the comprehensive and coherent design of the underlying management and continuity within the management approach. Against the background of a pursuit of consistent care, management continuity and individual control by the patient cover availability of the system and the ability of the care network to respond to the dynamic needs of the patients [62].

In order to achieve this goal, various management approaches can be used within the framework of coordinated care: conceivable options include, for instance, case management approaches or gate-keeping approaches [45].

The sub-dimension ‘Personalised Services’ describes the scope in various areas of decision-making and participation that is generally within the therapeutic limitations. Personalised or individualised care can be defined as care which takes account of the needs and expectations of the patients [47].

The dimension also describes the customisation of care processes to the needs and expectations of patients [31,38,46]. In terms of a multifactorial perspective, patient-centred design of healthcare and the associated recognition and consideration of the interdependencies of intellect, body and soul within the framework of coordinated care structures, prevention, health promotion and the long-term care of chronically ill patients are further decisive components of effective healthcare.

Finally, the dimension of ‘Social Participation’ describes the ability of patients not only to maintain their normal life within the community but also to continue their working life or resume participation as soon as possible during therapy. It is particularly important that the care is tailored to the patient's specific environment and that individual needs are taken into consideration. A positive social environment by friends and relatives [28,29,31] to motivate and support patients is one of the decisive success factors for (sustained) change of lifestyle habits and the maintenance of therapy rules [36].

## Conclusion

Delivering efficient healthcare within limited budgets requires an understanding of patient priorities. The costs in the healthcare system are increasing. However, expert judgements and patient needs or preferences are not always congruent [63] and hence treatment recommendations of healthcare providers are not always accepted and implemented by patients in a way which is therapeutically ideal or desirable. Against this background aligning clinical practice and health policy with patient priorities and the needs will improve the effectiveness of health interventions. Political decision-makers and medical healthcare providers should be enabled to make their decision on reliable information on expectations patients have on their treatment and whether a particular care programme is useful and sensible in relation to patient needs and preferences.

Andersen calls the individual requirements which, alongside other factors, decisively influence the decision to utilise healthcare services ‘needs’ [64] This term is used to describe all those factors which directly influence the decision to utilise services. Besides the subjective component, ‘perceived need’, this also includes the objective need established by experts, ‘evaluated need’. Andersen assumes that need represents the direct reason for utilising healthcare services [64]. Taking this a step further, it can be argued that an individual's motivation to utilise a healthcare offer is also considerably influenced by his preconceptions on health, which represent part of his predisposing characteristics. These individual ‘health beliefs’ have been described theoretically many times (see for instance, the ‘Health Belief Model’).

The patient is directly affected and should be focus of care. Patient-centred and innovative healthcare networks thus aim to make patient healthcare more effective and tailor the offers available to patient expectations. The initiation of suitable offers requires consideration of individual motivation and need conditions. A healthcare provider can motivate patients particularly well if he knows the factors which influence perceived patient utility. Healthcare offers and treatment processes should for this reason be tailored to patient priorities and expectations. If these are known, the efficacy of treatments and services can be improved and offers and services tailored to the individual needs of the patients.

### Limitations

This study could reveal seven patient-relevant dimensions of organised healthcare delivery systems that should be considered when designing new care programmes.

Even though the research team tried to recruit a balanced and representative sample especially the patient sample does not fulfil the criterion of representativity in terms of age. The mean age of the patients is 48.47 years and therefore much higher than the German mean age [65]. Moreover, the study represents cross-sectional data from 670 respondents from three German regions. Thus, a statement on the deviation from a representative norm sample is not possible.

The rating was based on a 5-point Likert scale. This survey technique has the inherent disadvantage that the individual items are not weighted against each other. Hence, respondents can rate each item as ‘very important’. The present study revealed this effect in the patient sample. Several items did not show a Gaussian distribution, but a left shift. A possible solution to that phenomenon would be the assessment with a method that is based on trade-offs between different characteristics. The latter technique allows the estimation of the relative importance of different aspects of care and the trade-offs between these aspects, while the direct assessment allows the inclusion of more aspects. Hence, both methods should be used in a combined way.

This study was based on the presumption: if healthcare services are tailored to the needs of the target group, it is assumed that the motivation to utilise those services and participate actively in therapy measures can be increased and long-term treatment success improved.

The results have created a precondition for an instrument to measure the effects of organised healthcare networks from the patient perspective. The extent to which the activation of patients through patient-centred care can improve the results of treatment remains to be seen. Therefore, the seven need dimensions were weighted in a follow-up project in the form of a Discrete Choice Experiment. This method aims to measure the influence of therapy characteristics on patient preferences [66]. Therefore, in a Discrete Choice Experiment, different therapies are presented pairwise and the subjects have to decide for one of the options [65,67].

## Figures and Tables

**Table 1.**
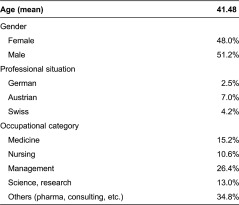
Socio-demographics: experts

**Table 2.**
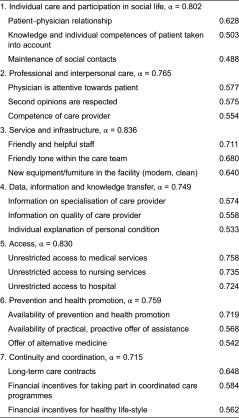
Results of factor analysis: experts

**Table 3.**
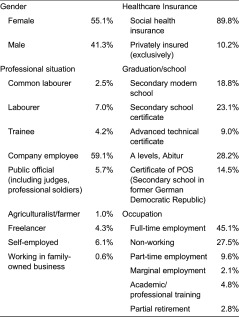
Socio-demographics: patients

**Table 4.**
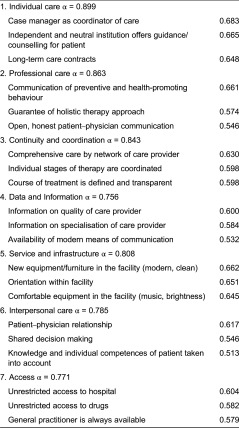
Results of factor analysis: patients

**Table 5.**
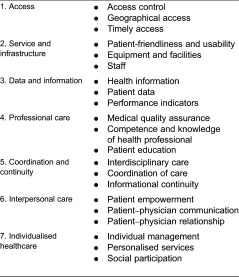
Model of needs dimensions
